# Die Europäische HNO-Facharztprüfung als hochwertiges Prüfungsinstrument: psychometrische Testkennwerte im Langzeitvergleich

**DOI:** 10.1007/s00106-025-01697-y

**Published:** 2026-01-22

**Authors:** Marcus Neudert, Aysenur Meric Hafiz, Victoria Ward, Ricard Simo, Wolfgang Luxenberger, Maria de la Mota, Cem Meco

**Affiliations:** 1 Klinik und Poliklinik für HNO-Heilkunde, Kopf- und Hals-Chirurgie, Universitätsklinikum Carl Gustav Carus, Technische Universität Dresden, Fetscher Str. 74, 01307 Dresden, Deutschland; 2 Hafiz Health Clinic, Hakkı Yeten Str. 10C/11 , Istanbul, Türkei; 3 Mid Yorks NHS Trust, Pinderfields Hospital, Wakefield, Großbritannien; 4 Guy’s and Saint Thomas’ Hospitals NHS Trust, London, Großbritannien; 5 EBEORL-HNS Administration, Madrid, Spanien; 6 Department of Otorhinolaryngology, Head and Neck Surgery, Ankara University Medical School, Ankara, Türkei; 7 Department of Otorhinolaryngology, Head and Neck Surgery, Salzburg Paracelsus Medical University, Salzburg, Österreich; 8 Department of Otolaryngology, Head and Neck Surgery, Cornell University, Weill Cornell Medical College, New York, USA

**Keywords:** Facharztprüfung, EBEORL-HNS, HNO-Heilkunde, SMP, Board exam, Specialty exam, EBEORL-HNS, Otorhinolaryngology head and neck surgery, SMP, Board exam

## Abstract

**Hintergrund:**

Die European Board Examination in Otorhinolaryngology – Head and Neck Surgery (EBEORL-HNS) stellt eine europaweit durchgeführte Facharztprüfung dar. Sie umfasst einen schriftlichen Multiple-Choice-Test und eine standardisierte mündliche Prüfung. Bisher liegen nur wenige Analysen zur psychometrischen Qualität dieser Prüfung vor.

**Ziel der Arbeit:**

Ziel war es, die Prüfungsdaten der letzten Jahre auszuwerten und die Prüfungsformate im Hinblick auf Schwierigkeit, Reliabilität und Bestehensquoten zu bewerten.

**Material und Methoden:**

Analysiert wurden aggregierte Jahreswerte (2013–2024) für Schwierigkeit, Trennschärfe, Cronbach‑α und Angoff-Grenzen, auch Mittelwert und Standardabweichung wurden berechnet. Schriftlicher und mündlicher Teil wurden getrennt und vergleichend ausgewertet.

**Ergebnisse:**

Die schriftliche Prüfung wies eine mittlere Schwierigkeit (Anteil korrekt beantworteter Items) von 67 % auf, das Cronbach‑α lag bei 0,83 (interne Konsistenz), und die Bestehensquote betrug im Mittel 82 %. Die Bestehensgrenze (per Angoff-Verfahren) lag im Mittel bei 59 Punkten. Die mündliche Prüfung zeigte eine mittlere Schwierigkeit von 70 %, eine Bestehensquote von 74 % und Mittelwerte von rund 3,0 Punkten auf einer vierstufigen Skala. Unterschiede zwischen den Jahren spiegelten v. a. die Zusammensetzung der internationalen Kandidatenkohorten wider.

**Schlussfolgerung:**

Die Daten zeigen, dass die EBEORL-HNS stabile Qualitätsmerkmale aufweist und im Vergleich zu nationalen Facharztprüfungen durch hohe Transparenz und definierte Qualitätskriterien hervorsticht. Unterschiede in den Bestehensquoten sind weniger Ausdruck von Defiziten des Prüfungsinstruments als Folge der heterogenen Zusammensetzung der Kandidatengruppen.

## Abstract (en)

**Background:**

The European Board Examination in Otorhinolaryngology, Head and Neck Surgery (EBEORL-HNS) is a Europe-wide specialty examination consisting of a written multiple-choice test and a standardized oral examination. Only limited psychometric analyses have been published to date.

**Objective:**

This study aimed to evaluate examination data from recent years and assess the formats regarding difficulty, reliability, and pass rates.

**Materials and methods:**

Aggregated data from 2013–2024 were analyzed. Mean scores, standard deviations, item difficulty, item discrimination, Cronbach’s alpha, and Angoff’s score were calculated. The written and oral parts were examined descriptively and comparatively.

**Results:**

The written examination demonstrated a mean difficulty of 67% (proportion of correctly answered items), a pass rate of 82%, and Cronbach’s alpha of 0.83 (internal consistency). The passing cutoff (according to the Angoff method) was 59 points on average. The oral part showed a mean difficulty of 70%, a pass rate of 74%, and an average score of around 3.0 on a four-point scale. Year-to-year variability largely reflected differences in the international candidate cohorts.

**Conclusion:**

The findings confirm that the EBEORL-HNS demonstrates stable psychometric quality. Compared to national specialty examinations, it offers high transparency and clearly defined quality criteria. Variations in performance appear more related to heterogeneous training backgrounds than to shortcomings of the assessment tools.

## Prüfverfahren

Die European Board Examination in Otorhinolaryngology – Head and Neck Surgery (EBEORL-HNS) ist ein europaweit standardisiertes Prüfverfahren mit dem Ziel, das Facharztwissen in Europa zu harmonisieren [1]. Grundlage bildet das UEMS-EBEORL-HNS Logbook als curricularer Referenzrahmen, der die inhaltliche Breite und Tiefe definiert und über einen Blueprint eine ausgewogene Repräsentation von Rhinologie, Otologie sowie Kopf-Hals-Chirurgie sicherstellt [2].

Die Prüfung besteht aus einem schriftlichen Multiple-Choice-Test mit 100 Items zur breiten Erfassung von Fakten- und Begründungswissen sowie einer nachgeschalteten standardisierten mündlichen Fallprüfung, die klinische Argumentation und Handlungsentscheidungen strukturiert abprüft. Für beide Komponenten liegen mehrjährige, systematisch erhobene Qualitätsdaten vor, die eine psychometrisch fundierte Bewertung von Schwierigkeit, Trennschärfe, Reliabilität und Standardsetzung ermöglichen.

Prüfungsqualität in der Medizin beruht auf transparenten Kennwerten auf Item- und Testebene. Die Itemschwierigkeit (Anteil korrekt beantworteter Fragen) bildet die Basis einer fairen Leistungsvermessung, während die Trennschärfe den Beitrag der Items zur Differenzierung zwischen stärker und schwächer performenden Kandidat:innen leistet. Auf Testebene weist die Reliabilität (Cronbach-α) die interne Konsistenz nach und ist zentral für die Interpretierbarkeit von Ergebnissen über Kohorten und Jahre hinweg. Für die Festlegung der Bestehensgrenze empfiehlt sich ein inhaltsbasiertes Standardsetzungsverfahren wie Angoff [4], das kohortenunabhängig und nachvollziehbar zu normativen Entscheidungen führt.

Im Sinne internationaler Gütekriterien erlauben diese Kennwerte ein kontinuierliches Qualitätsmonitoring und liefern Feedback für Curricula und Prüfungsdesign. Die vorliegende Arbeit berichtet über genau diese Parameter im Langzeitvergleich und macht deren Entwicklung für die europäische HNO-Facharztprüfung sichtbar.

Vor dem Hintergrund international heterogener Weiterbildungspfade bietet die EBEORL-HNS damit einen transparenten, empirisch anschlussfähigen Maßstab zur Beurteilung fachärztlicher Kompetenz.

## Material und Methoden

Die Arbeit basiert auf einer retrospektiven Längsschnittanalyse aggregierter Prüfungsdaten der European Board Examination in ORL-HNS. Dabei wurden die schriftlichen Prüfungen der Jahre 2013–2024 und der mündlichen Prüfungen der Jahre 2015–2024 analysiert. Aufbau und Ablauf der schriftlichen und mündlichen Prüfung sind bereits ausführlich beschrieben worden und werden hier nur für das Verständnis an relevanter Stelle erläutert [1, 3].

Es wurden gängige Kennzahlen der Prüfungsanalyse erhoben und u. a. Aussagen zur Schwierigkeit, Trennschärfe, Reliabilität, Bestehensgrenze und den Teilgebieten getroffen sowie Veränderungen im Zeitverlauf systematisch untersucht.

### Datenbasis

Schriftliche Prüfung: Die Auswertung basiert auf Prüfungsdaten aus 12 aufeinanderfolgenden Jahrgängen, die jeweils standardisiert aus 100 Einzel-Items bestanden (MC-Fragen). Jedes Item wurde einem der 3 Teilgebiete Rhinologie (*n* = 33), Otologie (*n* = 33) und HNS (*n* = 34) zugeordnet.

Mündliche Prüfung: Die Auswertung basiert auf Prüfungsdaten aus 9 Jahrgängen (2015 bis 2019, 2021 bis 2024), die jeweils aus 12 Items, hier standardisierten mündlichen Prüfungsfällen (SMP) (4 SMP je Teilgebiet Rhinologie, Otologie und HNS) bestanden. Im Jahr 2020 wurde pandemiebedingt keine mündliche Prüfung in Präsenz abgehalten, dafür 2021 eine mündliche Prüfung im Online-Verfahren.

### Erhobene Qualitätskennzahlen der schriftlichen Prüfung

#### Schwierigkeit

Die Schwierigkeit wurde als Anteil der korrekt beantworteten Items über alle Teilnehmenden berechnet. Sie wird hier als prozentualer Mittelwert (0–100 %) angegeben. Ein höherer Wert entspricht einer leichteren Prüfung. Für Items wird eine mittlere Schwierigkeit zwischen 40 und 85 % angestrebt. Zur Beschreibung des Niveaus und der zeitlichen Entwicklung wurden die Jahrgangsmittelwerte verglichen und Gruppenvergleiche durchgeführt.

#### Trennschärfe

Die Trennschärfe wurde als Item-Total-Korrelation erhoben, d. h. als Korrelation zwischen der Leistung in einem Item und der Gesamtleistung der jeweiligen Prüfung. Sie wurde ebenfalls auf aggregierter Ebene als Mittelwert/Jahrgang berechnet. Werte > 0,2 gelten als akzeptabel, Werte über 0,25 als gut. Die Trennschärfe erlaubt Aussagen darüber, ob Items geeignet sind, zwischen leistungsstarken und -schwachen Kandidat:innen zu differenzieren.

#### Bestehensgrenze

Die Bestehensgrenze (Passmark) wurde für jedes Jahr durch ein Angoff-Verfahren festgelegt. Dabei schätzen Fachexpert:innen für jedes Item, ob eine fiktive „gerade noch kompetente Person“ es richtig lösen würde. Der Mittelwert dieser Schätzwerte ergibt die Bestehensgrenze [4]. In dieser Studie wurde der Mittelwert der Angoff-Grenzen über alle Jahre berechnet und mit den jeweiligen Prüfungsergebnissen verglichen.

#### Bestehensquote

Aus den Angaben zu bestandenen und nicht bestandenen Prüfungen pro Jahr wurde die jeweilige Bestehensquote berechnet und in Prozent ausgedrückt. Dieser Wert gibt Aufschluss über die Durchlässigkeit der Prüfung und wurde im Kontext der Schwierigkeits- und Grenzwertanalysen interpretiert.

#### Reliabilität

Die interne Konsistenz der Klausuren wurde anhand des Cronbach‑α erfasst. Dieser Kennwert beschreibt die Zuverlässigkeit einer Prüfung und gibt an, ob die Items ein gemeinsames Konstrukt messen. Werte zwischen 0,80 und 0,90 gelten im Kontext hochschulischer Summativprüfungen als sehr gut. Für jede Klausur wurde der Wert auf aggregierter Ebene dokumentiert und in die vergleichende Auswertung aufgenommen.

#### Subgruppenvergleiche

Um den Einfluss struktureller Prüfungsformate zu untersuchen, wurden die Prüfungen in 2 Gruppen unterteilt: Präsenzprüfungen (2013–2019) und Online-Prüfungen (2020–2024). Diese Gruppen wurden im Hinblick auf Schwierigkeit, Bestehensgrenze, Trennschärfe, Reliabilität und Bestehensquote mittels deskriptiver Statistik und t‑Test für unabhängige Stichproben verglichen.

#### Teilgebietsspezifische Analyse

Zusätzlich wurden die Teilbereiche *Rhinologie, Otologie* und *HNS* getrennt ausgewertet. Für jedes Teilgebiet wurden die jährlichen Mittelwerte von Schwierigkeit und Trennschärfe erfasst und über den Gesamtzeitraum analysiert. So konnten fachspezifische Muster identifiziert werden, etwa in Bezug auf Item-Qualität und Differenzierungsfähigkeit.

### Erhobene Qualitätskennzahlen der mündlichen Prüfung

Die Schwierigkeit wurde als Anteil der korrekt beantworteten SMP über alle Teilnehmenden berechnet und als prozentualer Mittelwert (0–100 %) des Jahrgangs angegeben. Da die Prüfenden vorgegebene Bewertungskriterien (mit einer Punktevergabe von 1 bis 4, mit 3 Punkten gibt die einzelne SMP als bestanden) nutzen, sind Trennschärfe und Cronbach‑α in diesem Format nur begrenzt interpretierbar. Hier wurde daher auf die mittlere Schwierigkeit der SMP und die mittlere erreichte Punktzahl/Jahrgang eingegangen. Analog zur schriftlichen Prüfung wurden die Teilbereiche Rhinologie, Otologie und HNS getrennt ausgewertet.

### Statistische Auswertung

Die deskriptiven Analysen (Mittelwerte, Standardabweichungen) sowie Varianzanalysen (ANOVA) und Post-hoc-Tests (Tukey-HSD) wurden mit Microsoft Excel 2010 (Microsoft Corporation, Redmond, WA, USA) und IBM SPSS Statistics 27 (SPSS Inc., Chicago, IL, USA) durchgeführt. Simulierte Punktverteilungen auf Basis von Mittelwerten und Standardabweichungen dienten zur Plausibilisierung der Skalenverteilungen. Alle Tests erfolgten bei einem Signifikanzniveau von *p* < 0,05.

## Ergebnisse

### Statistische Analyse der schriftlichen Prüfungen

#### Schwierigkeit

Die schriftlichen Examina der Jahre 2013 bis 2024 zeigten über den gesamten Zeitraum hinweg ein vergleichbares Schwierigkeitsniveau. Der Mittelwert lag bei 67,4 %. Die Werte variierten dabei zwischen etwa 61 % (2013 und 2023) und über 71 % (2020 und 2021), Abb. 1.

**Abb. 1:**
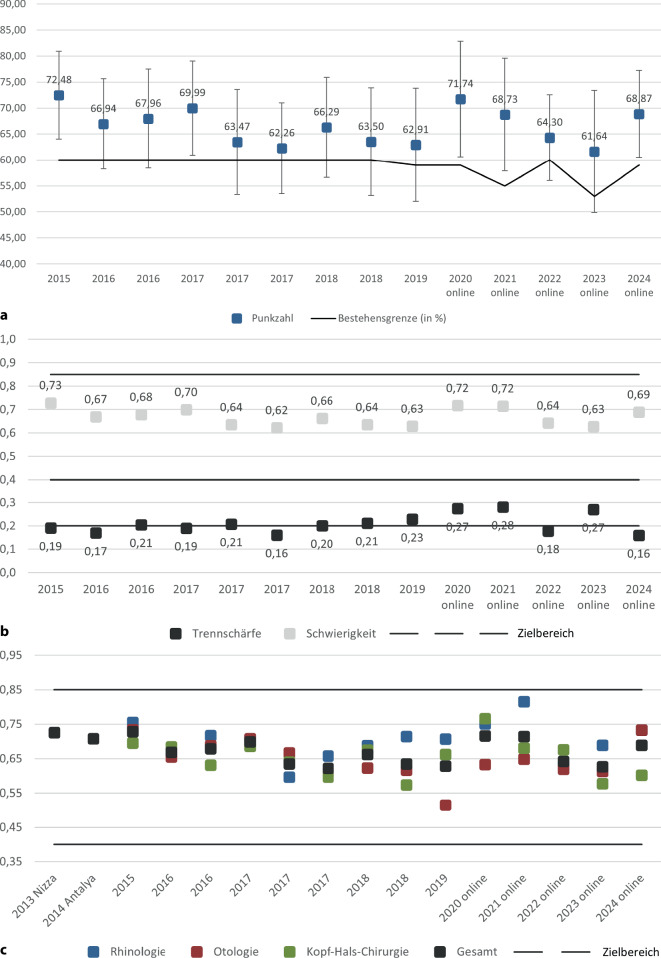
Qualitätsindizes der schriftlichen Prüfung. **a** Mittlere erreichte Punktzahl (Mittelwert ± SD) über die Jahre und Bestehensgrenze (aus dem Angoff-Verfahren). **b** Schwierigkeit und Trennschärfe über die Jahre mit Angabe der Zielbereiche für beide Indizes; **c** Darstellung der Schwierigkeit für die gesamt Prüfung und getrennt nach den Teilgebieten

#### Bestehensgrenze und Bestehensquote

Die Bestehensgrenze lag im Mittel bei 59 Punkten (auf der Originalpunktskala) (Abb. 1a). Der durchschnittliche Abstand zwischen der erreichten durchschnittlichen Punktzahl (≈67,4) und der Bestehensgrenze (≈59,1) betrug rund 8,3 Punkte. Dieser Abstand erscheint didaktisch angemessen und zeigt ein ausbalanciertes Verhältnis zwischen Durchlässigkeit und Selektion. Die mittlere Bestehensquote lag bei 82,1 %. Dies legt nahe, dass die Klausur über die Jahre hinweg für die Mehrheit der Kandidat:innen lösbar, aber keineswegs trivial war.

#### Trennschärfe

Die Trennschärfe lag im Mittel bei 0,208. Dieser Wert gilt im Kontext hochschulischer Prüfungen als gut, insbesondere bei einer Prüfung mit 100 Items, die ein breites Spektrum an Fachwissen abdeckt.

Einzelne Jahrgänge (z. B. 2021 und 2023) erreichten überdurchschnittliche Werte von > 0,25, während andere (u. a. 2015 oder 2024) darunter lagen (Abb. 1b).

#### Reliabilität

Die interne Konsistenz der Klausuren, gemessen mit dem Cronbach‑α, lag im Mittel bei 0,825. Dies ist ein sehr guter Wert, der auf eine hohe Zuverlässigkeit und Homogenität der Prüfung hinweist. Werte über 0,80 gelten in der hochschuldidaktischen Literatur als Indikator dafür, dass die Prüfung in sich stimmig ist und die Konstruktion der Items einem klaren Anforderungsprofil folgt (Tab. 1).

**Tab. 1:** Kennzahlen der schriftlichen Prüfungen im Vergleich des Durchführungsmodus

Kennzahl	Präsenz (2013–2019)	Online (2020–2024)
Ø Schwierigkeit (%)	67,24 %	67,83 %
Ø Trennschärfe	0,196	0,233
Ø Bestehensgrenze (Punkte)	59,91	57,20
Ø Cronbach‑α	0,818	0,840
Bestehensquote (%)	80,6 %	89,8 %

#### Teilgebietsanalysen: Rhinologie, Otologie, HNS

Das schriftliche Examen umfasst die 3 Hauptthemenbereiche Rhinologie, Otologie und HNS (Abb. 1c).

##### Rhinologie.

Die Rhinologie war im Mittel das leichteste Teilgebiet, mit einer durchschnittlichen Schwierigkeit von 70,3 %. In einzelnen Jahrgängen, etwa 2021, wurden Werte von über 81 % erreicht. Nur 2022 und 2023 lagen unter 70 %, aber auch hier blieb der Schwierigkeitsindex über 63 % (Mittelwert der Gesamtklausur aller Examina).

Die Trennschärfe war im Vergleich eher gering, im Mittel bei 0,197, was unterhalb des Schwellenwerts für sehr gute Item-Differenzierung liegt. Dies legt nahe, dass viele Fragen zwar richtig beantwortet wurden, aber nicht effektiv zwischen leistungsstarken und -schwachen Kandidat:innen unterscheiden konnten.

##### Otologie.

Die Otologie war das anspruchsvollste Teilgebiet, mit einer durchschnittlichen Schwierigkeit von 64,8 %. Besonders niedrig lag der Wert im Jahr 2023 (61,3 %), während 2024 mit 73,3 % den Höchstwert markierte. Die Spannweite ist hier also deutlich größer als in den anderen Subbereichen.

Gleichzeitig zeigte die Otologie die beste durchschnittliche Trennschärfe aller 3 Teilgebiete von 0,220. Es ist damit nicht nur fordernder, sondern auch besonders geeignet zur Leistungsdifferenzierung. In den Jahren 2020 und 2023 wurden sehr gute Trennschärfen (> 0,25) erzielt, was für die Qualität der Fragen in diesem Teilbereich spricht.

##### HNS.

Die Kopf-Hals-Chirurgie lag in der Schwierigkeit im mittleren Bereich mit durchschnittlich 65,4 %. Die Variation war auch hier ausgeprägt: von einem Höchstwert in 2020 (76,6 %) bis zu einem Tiefstand in 2023 (57,8 %).

Die Trennschärfe betrug im Mittel 0,212, was ähnlich wie in der Otologie einem guten Differenzierungsniveau entspricht. Besonders leistungsstark war die HNS-Differenzierung im Jahr 2020 (0,296) und 2021 (0,259), während das Jahr 2024 signifikant geringer war.

#### Vergleich von schriftlichen Online- und Präsenzprüfungen

Im Zuge der COVID-19-Pandemie wurden ab dem Jahr 2020 die schriftlichen Prüfungen im Online-Format durchgeführt. Um potenzielle Auswirkungen dieser Umstellung auf Prüfungsleistung und Gütekriterien zu untersuchen, wurden die Jahrgänge 2013–2019 (Präsenz) mit den Jahrgängen 2020–2024 (Online-Format) verglichen (Abb. 1a–c).

Die durchschnittliche Schwierigkeit lag in den Online-Prüfungen bei 67,8 %, gegenüber 67,2 % in den Präsenzprüfungen. Auch die Bestehensgrenze, die jeweils auf Basis eines Angoff-Verfahrens festgelegt wurde, war in den Online-Jahren im Schnitt niedriger (57,2 Punkte) als in der Präsenzphase (59,9 Punkte).

Die Bestehensquote war in den Online-Jahren mit 89,8 % deutlich höher als in den Präsenzjahren (80,6 %). Gleichzeitig wiesen die Online-Prüfungen auch eine höhere Trennschärfe (Ø 0,233 vs. 0,196) auf – ein Hinweis darauf, dass trotz des leichteren Formats eine gute Differenzierung zwischen leistungsstärkeren und -schwächeren Kandidat:innen möglich war.

Hinsichtlich der Reliabilität zeigten sich in den Online-Jahren ebenfalls leicht höhere Werte: Das Cronbach‑α lag im Mittel bei 0,840, während es in den Präsenzprüfungen 0,818 betrug, was für in beiden Fällen eine hohe interne Konsistenz anzeigt.

### Statistische Analyse der mündlichen Prüfungen

#### Erreichte Punktzahl, Schwierigkeit und Bestehensquote

**Abb. 2:**
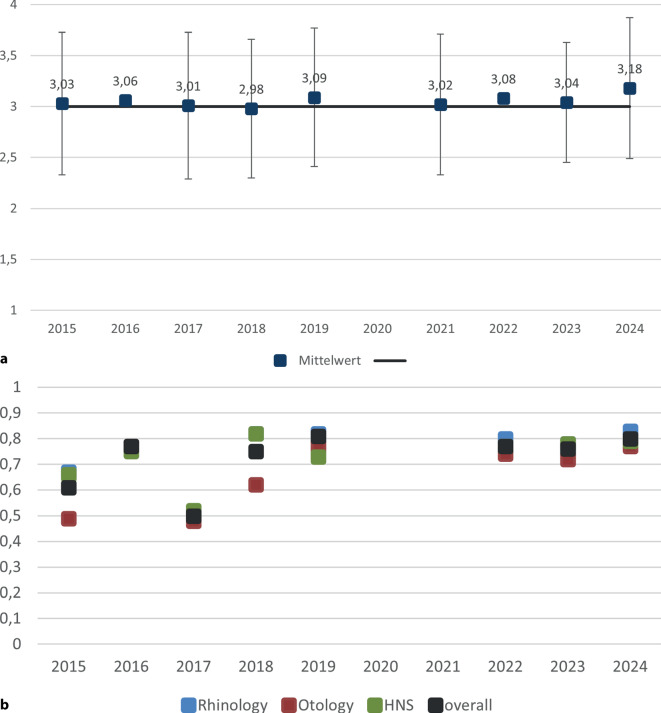
Qualitätsindizes der mündlichen Prüfung. **a** Mittlere erreichte Punktzahl (Mittelwert ± SD) über die Jahre und Gesamtmittelwert (3,04 Punkte) für die Gesamtprüfung; **b** Schwierigkeit gesamt und nach Teilgebieten über die Jahre. Im Jahr 2020 pandemiebedingt keine Prüfung, im Jahr 2021 eine mündliche Online-Prüfung, wobei eine stratifizierte Auswertung nicht möglich war

Die durchschnittlich erreichte Punktzahl lag über alle Jahrgänge hinweg bei 3,04 (Abb. 2a). Dabei ist zu berücksichtigen, dass das Bewertungssystem ausschließlich ganzzahlige Punktwerte zwischen 1 und 4 vorsieht, die nicht linear mit klassischen Schulnoten vergleichbar sind. Ein Punktwert von 3 zeigt eine kompetente Standardleistung (bestanden) an. Vor diesem Hintergrund ist ein Mittelwert von 3,04 so zu interpretieren, dass die Kandidat:innen im Mittel knapp über dem Bestehensniveau lagen, mit einer leichten Tendenz zur soliden Beherrschung der Inhalte. Anders als in linearen Notenskalen bedeutet eine Punktdifferenz von ±0,3 in diesem System bereits eine spürbare qualitative Abstufung.

Die Schwierigkeit, berechnet als Anteil der maximal erreichbaren Punktzahl (relativ zu 4 Punkten), betrug im Mittel 70,0 %, was im Kontext des Bewertungsmodells einem gut lösbaren, aber nicht trivialen Anforderungsniveau entspricht. Die Standardabweichung von 0,70 Punkten zeigt, dass die Prüfung in der Lage war, Leistungsunterschiede zuverlässig abzubilden.

Die durchschnittliche Teilnehmerzahl pro Jahrgang betrug 123 Personen.

Die durchschnittliche Bestehensquote lag bei 74,0 %, d. h., etwa ein Viertel der zugelassenen Kandidat:innen bestand die mündliche Prüfung nicht. Im Vergleich zur schriftlichen Prüfung (Ø 82,1 %) stellt die mündliche Prüfung damit ein deutlich selektiveres Element des Gesamtverfahrens dar.

#### Teilgebietsanalyse

Ein Vergleich der 3 geprüften Teilgebiete zeigt deutliche Unterschiede (Abb. 2b):

*Rhinologie* war das leichteste Teilgebiet mit einer durchschnittlichen Schwierigkeit von 74,0 % und dem höchsten Punktwert (Ø 3,25).

*Otologie* stellte die höchsten Anforderungen: Die Schwierigkeit lag im Mittel nur bei 65,6 %, die erreichte Punktzahl bei 3,02.

*Kopf-Hals-Chirurgie (HNS)* lag mit 72,7 % Schwierigkeit und einem Punktwert von 3,03 zwischen den beiden anderen Bereichen.

#### Vergleich von mündlichen Online- und Präsenzprüfungen

Im Jahr 2021 wurde pandemiebedingt die mündliche Facharztprüfung einmalig im Online-Format durchgeführt. Der Mittelwert der Gesamtleistung betrug 3,08 Punkte und entsprach damit sehr genau dem Durchschnittswert der übrigen Jahre (Abb. 2a). Eine Berechnung der Schwierigkeit war aufgrund der Datenübermittlung durch den kommerziellen Anbieter nicht möglich. In der fachlichen Differenzierung zeigte sich ein vertrautes Muster: Rhinologie erzielte mit 3,62 Punkten den höchsten Wert, während Otologie mit 2,94 Punkten etwas niedriger lag und Head and Neck mit 3,09 Punkten im Bereich des Gesamtdurchschnitts blieb. Insgesamt deuten die Ergebnisse darauf hin, dass die Online-Durchführung trotz des außergewöhnlichen Formats vergleichbare Resultate wie die Präsenzprüfungen hervorbrachte und keine systematischen Verzerrungen erkennbar sind.

## Diskussion

Die Ergebnisauswertung der European Board Examination in Otorhinolaryngology – Head and Neck Surgery über die Jahre 2013 bis 2024 erlaubt eine differenzierte Betrachtung zentraler Qualitätsmerkmale einer europaweit durchgeführten Facharztprüfung. Bemerkenswert ist zunächst die Verfügbarkeit systematisch erhobener Prüfungskennzahlen über einen langen Zeitraum, was für hochschul- und weiterbildungsdidaktische Fragestellungen eine robuste Grundlage schafft.

### Die europäische Facharztprüfung ist eine hochwertige Prüfungsform mit dokumentierten Qualitätsindizes

Die schriftliche Komponente besteht aus einem Multiple-Choice-Test mit 100 Items, überwiegend Typ A und einem (etwa 10 %igen) Anteil Kprim-Fragen. Die mündliche Komponente folgt einem standardisierten Prüfungsparcours mit strukturierten Fällen (SMP), die in festen Zeitfenstern geprüft werden. Beide Teile sind über einen Blueprint inhaltlich balanciert und auf eine breite Repräsentativität des Fachs ausgelegt, sodass alle 3 großen Teilbereiche Rhinologie, Otologie und Kopf-Hals-Chirurgie in vergleichbarer Gewichtung berücksichtigt sind. Die schriftliche Prüfung bildet in der Sequenz den ersten Abschnitt, zur mündlichen werden nur Kandidat:innen zugelassen, die den schriftlichen Teil bestanden haben. Diese Abfolge etabliert eine vorgelagerte Schwelle und gewährleistet, dass in der mündlichen Prüfung ein Mindestniveau an fachlichem Wissen vorausgesetzt werden kann.

Die Analyse der globalen Schwierigkeit, der Trennschärfe und der Reliabilität zeigt durchgängig Werte im Bereich guter psychometrischer Qualität. Der mittlere Schwierigkeitsindex der schriftlichen Prüfung lag über alle Jahre bei etwa 67 %, mit erwartbaren Schwankungen zwischen Jahrgängen. Dass Items im mittleren Schwierigkeitsbereich die größte Differenzierungsleistung entfalten, ist in der messtheoretischen Literatur gut belegt, und die beobachtete Verteilung der Trennschärfen entspricht diesem Befund. Die interne Konsistenz der schriftlichen Prüfung war mit einem durchschnittlichen Cronbach‑α von 0,825 hoch, was auf eine kohärente Itemsammlung hindeutet, die ein gemeinsames Konstrukt zuverlässig abbildet [5]. Die Bestehensgrenze wurde jährlich nach einem modifizierten Angoff-Verfahren festgelegt, im Mittel bei rund 59 Punkten. Dieses Vorgehen ist international etabliert, setzt aber ein sorgfältiges Expertengremium, Prüfertraining und Ankerbeispiele voraus, damit die Schwelle stabil und nachvollziehbar bleibt [6].

Die Teilgebietsanalysen geben zusätzliche Hinweise auf die inhaltliche Architektur der Prüfung. Rhinologie war im schriftlichen Teil im Mittel am leichtesten, zeigte aber die geringste mittlere Trennschärfe. Otologie war anspruchsvoller und wies die beste Differenzierungsleistung auf. Kopf-Hals-Chirurgie lag zwischen beiden Bereichen. Diese Muster deuten nicht auf systematische Ungleichgewichte hin, vielmehr auf unterschiedliche kognitive Anforderungsprofile, die sich in der Bearbeitungstiefe und der Art der Schlussfolgerungen niederschlagen. Auffällig ist, dass einzelne Jahrgänge durchgängig schwächere Trennschärfen aufwiesen, was im Kontext der Kohortenstruktur und möglicher externer Einflüsse interpretiert werden sollte. Eine rein prüfungsbezogene Erklärung wäre zu kurz gegriffen, wenn in denselben Jahren auch die Zusammensetzung der Teilnehmenden oder die Vorbereitungspraxis Veränderungen aufweist.

### Prüfungsaufbau und -struktur erhöhen die Prüfungsqualität

Die mündliche Prüfung ist in Aufbau und Zielsetzung komplementär zur schriftlichen. Sie prüft in strukturierter Form klinisches Handeln, begründetes Entscheiden und kommunikative Darlegung [7–9]. Das Bewertungssystem arbeitet mit einer vierstufigen Skala, wobei 3 Punkte einem kompetenten Standard entsprechen, 4 Punkte eine überdurchschnittliche Leistung anzeigen, 2 Punkte einen knapp nicht ausreichenden Befund markieren und ein Punkt eine unzureichende Leistung kennzeichnet. Ein mittlerer Punktwert von etwa 3,0 ist in diesem System daher nicht als Mittelmaß im umgangssprachlichen Sinn zu lesen, sondern als stabile Leistung oberhalb der Bestehensgrenze. Die Schwierigkeit der mündlichen Prüfung lag im Mittel bei etwa 70 %, die Bestehensquote bei rund 74 %. Im Vergleich zur schriftlichen Prüfung ist dies eine selektivere Hürde, was mit der stärkeren Gewichtung von Anwendung, Begründung und fallbezogener Entscheidung kongruent ist. Dass die Varianz der Punktwerte im mündlichen Teil geringer ausfällt als in breit streuenden Prozentpunktskalen, ist systemimmanent und erschwert einfache Vergleiche über Skalen hinweg. Die Standardisierung der Fälle, der Zeitfenster und der Bewertungsraster ist dabei zentral, um Objektivität und Vergleichbarkeit zu sichern [7, 10].

Ein zentrales Ergebnis der getrennten und gemeinsamen Betrachtung ist, dass die beiden Formate unterschiedliche, aber sich ergänzende Kompetenzdimensionen adressieren. Die schriftliche Prüfung erfasst effizient breites Fakten- und Basisbegründungswissen mit hoher Stichprobenbreite über das Fachgebiet. Die mündliche Prüfung nimmt klinische Entscheidungsfindung und -begründung, Priorisierung und Handlungsplanung in den Blick. Im Sinne einer Triangulation entstehen dadurch 2 sich wechselseitig stützende Evidenzlinien für die Leistungsbeurteilung [11]. Die höheren Reliabilitätswerte des schriftlichen Formats bilden die Stärken standardisierter Wissensmessung ab, die Vertiefung in Fallszenarien der mündlichen Prüfung spiegeln den Anspruch wider, auch die Umsetzung in klinisch-praktische Entscheidungen zu prüfen. Genau hier liegt eine Stärke der EBEORL-HNS, die diese beiden Ansprüche durch Kombination beider Prüfungsanteile verbindet. Dies sichert eine Balance zwischen Reliabilität und Validität, die in vielen rein schriftlichen oder rein mündlichen nationalen Prüfungen, wie auch in Deutschland, nicht erreicht wird [12]. Der beobachtete Unterschied der Bestehensquoten ist vor diesem Hintergrund plausibel und kein Hinweis auf ein Ungleichgewicht, sondern Ausdruck getrennter Zielsetzungen innerhalb der gesamten Prüfung.

Die Auswertung der Jahre mit Online-Durchführung der schriftlichen Prüfung zeigt keinen Leistungsnachteil gegenüber Präsenzformaten. Die durchschnittliche Schwierigkeit war in den Online-Jahren leicht höher (die Prüfung gering leichter), die Trennschärfen tendenziell besser und die Reliabilität mindestens gleichwertig. Die Bestehensgrenze lag im Mittel etwas niedriger. Zusammen genommen sprechen diese Kennzahlen nicht für einen systematischen Moduseffekt, sondern für eine gelungene Übertragung des Formats. Dass sich die Bestehensquote in den Online-Jahren erhöhte, ist bei gleichzeitiger Aufrechterhaltung guter Trennschärfen kompatibel mit einer moderaten Erleichterung auf Itemebene, ohne Selektivität einzubüßen. Dies ist ein Befund, der in internationalen Vergleichen digitaler Prüfungsumstellungen wiederholt beobachtet wurde, sofern Blueprint, Standardsetzung und statistische Kontrolle konsequent umgesetzt werden.

### Kohortenzusammensetzung und Weiterbildungscurricula der Herkunftsländer haben Einfluss auf das Prüfungsergebnis

Die jährlichen Schwankungen in Schwierigkeit, Trennschärfe und Bestehensquote sind insbesondere vor dem Hintergrund der inhomogenen Kandidatenschaft zu interpretieren. An der Prüfung nehmen Ärztinnen und Ärzte aus verschiedenen Ländern teil, mit unterschiedlichen Weiterbildungsdauern, nationalen Curricula und klinischen Erfahrungsprofilen. Diese strukturelle Vielfalt prägt die Kohorten. Sie kann dazu führen, dass sich Jahrgänge nicht allein durch das Prüfungsinstrument unterscheiden, sondern auch durch Vorbereitungserfahrungen, sprachliche Kontexte, Prüfungskultur und Fallvertrautheit. Obwohl die UEMS für alle Fachgebiete europäische Mindestanforderungen formuliert hat, ist die tatsächliche Umsetzung in den Ländern und auch den Weiterbildungsstätten unterschiedlich. Diese sind in nicht EU-Staaten teilweise noch stärker ausgeprägt. Die strukturelle Inhomogenität der Weiterbildung stellt somit einen großen Einflussfaktor auf die Prüfungsergebnisse dar und muss bei der Interpretation der Prüfungsstatistiken mitgedacht werden. Internationale Arbeiten zu standardisierten medizinischen Prüfungen verweisen zudem darauf, dass Unterschiede in Ausbildungssystemen und Prüfungsgewohnheiten messbar sind und sich in Ergebnissen widerspiegeln können, ohne dass daraus ein Qualitätsdefizit des Instruments abzuleiten wäre. Auswertungen von medizinischen Examina, etwa in den USA oder Kanada, zeigen, dass Ärzt:innen mit einem internationalen Ausbildungsweg abweichende Leistungen in standardisierten Prüfungen erzielen, weil Struktur, Prüfungsformate und Schwerpunkte ihrer Ausbildung nicht immer mit den Anforderungen der durchgeführten Prüfung übereinstimmen [13, 14]. Für die Interpretation der Ergebnisse bedeutet dies, dass statistische Kennziffern stets in den Kontext der Kohorte gestellt werden sollten. Wo möglich, sind stratifizierte Auswertungen nach Herkunftsregionen, Ausbildungslinien oder Vorbereitungspfaden hilfreich, um plausible Erklärungen für beobachtete Muster zu gewinnen. Solche Analysen wurden bisher nicht vorgenommen, können aber in der Zukunft noch mehr Verständnis für die Unterschiede der nationalen Weiterbildungssysteme generieren.

### Stellung der europäischen Facharztprüfung in EU-Mitgliedsstaaten und Deutschland

Die Analyse der europäischen Facharztprüfung lässt sich in ein breiteres internationales Umfeld einordnen, in dem eine starke Heterogenität der Facharztprüfungspraxis besteht. Viele nationale Prüfungen sind wenig standardisiert und insbesondere kompetenzorientierte Dimensionen wie Kommunikation oder klinisches Handeln nur unzureichend abgebildet [7, 15]. International empfohlen wird eine Methoden-Triangulation aus schriftlichen, mündlichen und praktischen Formaten, ergänzt durch verpflichtendes Blueprinting, strukturierte Erwartungshorizonte, Prüfer:innentrainings, ausreichende Aufgaben- und Prüferzahlen sowie teststatistische Qualitätssicherung [7]. Vor diesem Hintergrund erscheint die EBEORL-HNS als ein Prüfungsinstrument, das europaweit eine Vorreiterrolle einnimmt, da es standardisierte, publizierte Qualitätskennwerte liefert und durch sein duales Format eine hohe Validität aufweist. Dass in einigen Ländern die europäische Prüfung bereits anerkannt ist, während Deutschland diese bislang nicht berücksichtigt, verdeutlicht die politischen Dimensionen der Anerkennungsfrage. Die Frage nach der Stellung der europäischen HNO-Facharztprüfung im Verhältnis zu nationalen Prüfungen ist in erster Linie eine Frage nach dem Grad an Transparenz, Standardisierung und empirischer Absicherung. Die hier vorliegenden Daten belegen, dass die Prüfung über viele Jahre konsistente Qualitätsindizes aufweist, die international üblichen Gütekriterien entsprechen und in ihren Zielsetzungen klar definiert sind. Der europäische Kontext zeigt, dass unterschiedliche Wege der Einbindung praktikabel sind. In mehreren Mitgliedstaaten wird die bestandene Prüfung als nationale Facharztprüfung anerkannt oder der nationalen Prüfung gleichgestellt, sodass Absolvent:innen und Absolventen die Qualifikation im Herkunftsland anrechnen lassen können. Die Schweiz nutzt den schriftlichen Teil als verbindliches Element (obwohl in englischer Sprache) und ergänzt ihn um eine national verantwortete mündlich-praktische Komponente. Diese Modelle belegen, dass eine partielle oder vollständige Integration möglich ist, wenn Zuständigkeiten klar beschrieben und Qualitätsanforderungen eindeutig kommuniziert werden. Sie illustrieren zudem, dass eine schrittweise Annäherung ohne Bruch bestehender Strukturen durch definierte Gleichwertigkeitsregelungen umsetzbar ist. Die Tab. 2 zeigt die aktuelle Anerkennungspraxis der europäischen Facharztprüfung in Europa (Tab. 2).

**Tab. 2:** Übersicht über die verschiedenen Modelle der Anerkennung der europäischen Facharztprüfung, European Board Examination of Otorhinolaryngology, Head and Neck Surgery (EBEORL-HNS), Stand: August 2025

EBEORL-HNS-Prüfung …	Staaten	Bemerkung
Ersetzt die nationale Facharztprüfung	Belgien, Island, Malta	Es gibt keine nationale Facharztprüfung, die europäische Prüfung ersetzt eine nationale
Ist äquivalent zur nationalen Facharztprüfung	Finnland, Griechenland, Kroatien, Schweden, Türkei	Weiterbildungsassistent:innen können entscheiden, welche Prüfung sie machen.
Ersetzt einen Teil der nationalen Facharztprüfung	Schweiz	Der schriftlicher Teil der europäischen Prüfung wird absolviert und durch den nationalen mündlich-praktischen ergänzt.

In Deutschland besteht bislang keine formale Anerkennung durch die Landesärztekammern. Diese Situation ist insofern bemerkenswert, als die europäische Prüfung eine Datentransparenz bietet, die im nationalen Kontext in dieser Breite nicht verfügbar ist oder nur nicht publiziert wird. Ob das europäische Examen in Deutschland Anerkennung finden wird, scheint demnach eher eine (berufs)politische Frage zu sein. Aus Sicht der Weiterbildungsassistent:innen stellt die europäische Prüfung bislang einen freiwilligen Qualifikationsnachweis und ein wertvolles Benchmarking im internationalen Vergleich dar. Der erfolgreiche Abschluss könnte klare Karrierevorteile ermöglichen, wenn Klinikdirektor:innen die Weiterbildungsassistent:innen gezielt zur Teilnahme motivieren und diese Zusatzqualifikation in Bewerbungsverfahren aktiv erfragen und berücksichtigen. Diese Entwicklung würde zusätzlich gestützt, wenn Fachgesellschaft und Berufsverband die Prüfung als hochrangiges Qualitätsinstrument wahrnehmen, bewerben und offiziell unterstützen. Dadurch entstünden Akzeptanz in der Fachöffentlichkeit und eine starke Signalwirkung, die entscheidend für die Diskussion mit den Ärztekammern auf Bundes- und Länderebene wären. Diese gilt es konstruktiv zu führen und voranzutreiben, um auch in Deutschland mittelfristig Modelle der Teilanrechnung oder Anerkennung zu etablieren. Auf diese Weise ließe sich die europäische Facharztprüfung als komplementäres Element in die deutsche Weiterbildungslandschaft integrieren und für die Weiterbildungsassistent:innen, das Fachgebiet und die Harmonisierung der Weiterbildung in Europa einen deutlichen Mehrwert schaffen.

## Fazit für die Praxis

- Die European Board Examination in Otorhinolaryngology – Head and Neck Surgery weist über viele Jahre hinweg stabile Qualitätsindikatoren auf.
- Schriftliche und mündliche Prüfung ergänzen sich hinsichtlich ihrer taxonomischen Niveaus und gewährleisten eine breit angelegte, valide Kompetenzbewertung.
- Unterschiede in Bestehensquoten oder Schwierigkeit sind weniger durch die Prüfungsinstrumente selbst als vielmehr durch die internationale Heterogenität der Kandidat:innen bedingt.
- Für Deutschland könnte die Anerkennung der Prüfung einen Beitrag zur internationalen Vergleichbarkeit leisten und wäre auch für Weiterbildungsassistent:innen sowie Kliniken und Weiterbildungsstätten ein relevanter Benchmark.
- Die Prüfung liefert nachvollziehbare Kennzahlen, die Transparenz und Qualität der Facharztprüfung dokumentieren.

## Data Availability

Die erhobenen Datensätze können auf begründete Anfrage in anonymisierter Form beim korrespondierenden Autor angefordert werden.
